# Letter to the Editor: “Adverse Events and Subsequent Management Associated With Eustachian Tube Balloon Dilation”

**DOI:** 10.1002/oto2.95

**Published:** 2023-11-22

**Authors:** Aashish Batheja, Daniel H. Coelho

**Affiliations:** ^1^ Department of Otolaryngology–Head & Neck Surgery Virginia Commonwealth University School of Medicine Richmond Virginia USA

1

We deeply appreciate the paper by Jeoung et al analyzing adverse events from balloon dilation of the eustachian tube (BDET) using the U.S. Food and Drug Administration's Manufacturer and User Facility Device Experience (MAUDE) database.^1^ Given the understudied nature of this topic, we agree that more research investigating complications of BDET will better inform clinical decision‐making.^1^ On May 8, 2023, we searched the MAUDE database for medical device reports involving BDET (indicated by product code “PNZ”) and gathered data on reported events and associated complications. Our independent investigation supports the major conclusions of Jeoung et al while offering additional clinical context and discussion of an adverse event reported after their search period.

Our investigation confirms the report of hearing impairment, although the mechanism by which BDET can result in hearing loss is unconfirmed. One possibility is damage to the round window from a large change in pressure due to improper catheter removal, while another theory is failure of the inner ear to adequately adjust to changes in static pressure.^2^ Regardless, proper use of the insertion tool can reduce hearing loss risk.^2^

Perhaps the most notable finding was the report of stroke attributed to right carotid artery injury, which calls for renewed discussion on imaging prior to BDET. Manufacturers such as Acclarent encourage imaging before BDET to address the risk of carotid artery injury, listing carotid artery dehiscence as a contraindication.^3^ One expert panel claimed that if a dehiscent carotid artery is noted on imaging, then the use of a depth marker during BDET is necessary.^4^ However, the same panel failed to reach consensus on whether CT imaging before BDET is essential.^4^ Arguments against preoperative CT imaging cite limited predictive ability for complications and increased radiation.^5^ The lack of agreement on this issue suggests further discussion is necessary to define the role of imaging prior to BDET. Future conversations should include consideration of the carotid artery injury reported in MAUDE.

Our investigation also reveals a report of bradycardia with the clinicians involved believing balloon dilation to be the cause but the manufacturer claiming that other factors like anesthesia could have contributed. The type of anesthesia used was not reported. What remains clear is, although imperfect, the MAUDE database can shed light on real‐world complications in the hands of end‐users, not only those in clinical trials by high‐volume experienced surgeons.

## Disclosures

### 1 Competing interests

None.

### 2 Funding source

None.
